# Subverted regulation of Nox1 NADPH oxidase-dependent oxidant generation by protein disulfide isomerase A1 in colon carcinoma cells with overactivated KRas

**DOI:** 10.1038/s41419-019-1402-y

**Published:** 2019-02-13

**Authors:** Tiphany Coralie De Bessa, Alessandra Pagano, Ana Iochabel Soares Moretti, Percillia Victoria Santos Oliveira, Samir Andrade Mendonça, Herve Kovacic, Francisco Rafael Martins Laurindo

**Affiliations:** 1 LIM 64, Instituto do Coracao (InCor), Hospital das Clinicas HCFMUSP, Faculdade de Medicina, Universidade de Sao Paulo, Sao Paulo, SP Brazil; 2 Aix Marseille Univ, CNRS, UMR 7051, INP, Inst Neurophysiopathol, Faculté de Pharmacie, 27, Boulevard Jean Moulin - 13385 Marseille CEDEX 5-France, Marseille, France; 3 Centro de Investigação Translacional em Oncologia do Instituto do Câncer do Estado de São Paulo (Icesp), Faculdade de Medicina, Universidade de Sao Paulo, Sao Paulo, SP Brazil

## Abstract

Protein disulfide isomerases including PDIA1 are implicated in cancer progression, but underlying mechanisms are unclear. PDIA1 is known to support vascular Nox1 NADPH oxidase expression/activation. Since deregulated reactive oxygen species (ROS) production underlies tumor growth, we proposed that PDIA1 is an upstream regulator of tumor-associated ROS. We focused on colorectal cancer (CRC) with distinct KRas activation levels. Analysis of RNAseq databanks and direct validation indicated enhanced PDIA1 expression in CRC with constitutive high (HCT116) vs. moderate (HKE3) and basal (Caco2) Ras activity. PDIA1 supported Nox1-dependent superoxide production in CRC; however, we first reported a dual effect correlated with Ras-level activity: in Caco2 and HKE3 cells, loss-of-function experiments indicate that PDIA1 sustains Nox1-dependent superoxide production, while in HCT116 cells PDIA1 restricted superoxide production, a behavior associated with increased Rac1 expression/activity. Transfection of Rac1^G12V^ active mutant into HKE3 cells induced PDIA1 to become restrictive of Nox1-dependent superoxide, while in HCT116 cells treated with Rac1 inhibitor, PDIA1 became supportive of superoxide. PDIA1 silencing promoted diminished cell proliferation and migration in HKE3, not detectable in HCT116 cells. Screening of cell signaling routes affected by PDIA1 silencing highlighted GSK3β and Stat3. Also, E-cadherin expression after PDIA1 silencing was decreased in HCT116, consistent with PDIA1 support of epithelial–mesenchymal transition. Thus, Ras overactivation switches the pattern of PDIA1-dependent Rac1/Nox1 regulation, so that Ras-induced PDIA1 bypass can directly activate Rac1. PDIA1 may be a crucial regulator of redox-dependent adaptive processes related to cancer progression.

## Introduction

Protein disulfide isomerase (PDI or PDIA1) is a dithiol/disulfide oxidoreductase chaperone from the endoplasmic reticulum (ER), where it assists redox protein folding and thiol isomerization. PDIA1 is the prototype of a multifunctional family having > 20 members^1,2^. In addition, PDIA1 is involved in redox cell signaling regulation at distinct levels^1^. PDIA1 can also locate at the cytosol, cell surface, and is secreted by distinct cell types^3^. Cell-surface/secreted PDIA1 regulates virus internalization, thrombosis, platelet activation, and vascular remodeling^1,4^. Overall, PDIA1 is implicated in the pathophysiology of cardiovascular and neurodegenerative disorders, diabetes, and, in particular, cancer^5^. Several PDIs such as PDIA1, PDIA6, PDIA4, and PDIA3 are reportedly upregulated in cancer^6^. PDIA1, in particular, is overexpressed in melanoma, lymphoma, hepatocellular carcinoma, brain, kidney, ovarian, prostate, and lung cancers^6–10^ and frequently associates with metastasis, invasiveness, and drug resistance^11,12^. Conversely, lower tumor PDIA1 levels associate with improved survival in breast cancer and glioblastoma^13^. In glial cells, breast and colorectal cancer, PDIA1 overexpression has been proposed as a cancer cell biomarker^13–15^. The mechanisms whereby PDIA1 supports tumor progression are yet poorly understood.

An important cancer cell hallmark is the enhanced output of reactive oxygen species (ROS) such as superoxide, hydrogen peroxide, peroxynitrite, etc., which engage into disrupted signaling routes that further support tumorigenesis or metastasis, but in some instances may suppress tumor propagation^16^. Such dual oxidant effects of ROS in tumorigenesis may underlie transition from adaptive to maladaptive responses enabling tumor escape^17^. Therefore, mechanisms of ROS regulation can illuminate the understanding of tumor biology and are potential therapeutic targets. Most of such mechanisms converge to enzymatic ROS sources, such as mitochondrial electron transport and Nox family NADPH oxidases. Noxes, in particular, have been increasingly implicated in cancer pathophysiology^18^. The upstream mechanisms governing Nox-dependent processes in cancer are not fully understood. In vascular cells, our group has shown consistent correlation between PDIA1 and Nox-dependent ROS generation. PDIA1 silencing/inhibition abrogates growth factor-dependent Nox1 activation and expression^19–21^ and, in parallel, significantly disrupts cytoskeletal organization, RhoGTPase activation, and cell migration^4,21^. Acute PDIA1 overexpression supports agonist-independent superoxide production and Nox1 expression in vascular smooth muscle (VSMC)^20,21^. PDIA1 similarly converges with Nox2 in phagocytes^22,23^. We propose that PDIA1 is a relevant upstream regulatory mechanism of ROS generation in tumor cells. Conversely, understanding mechanisms associated with PDIA1/Nox convergence may help to understand the roles of PDIA1 in cancer pathophysiology.

Here, we focused on colorectal cancer cells (CRC), since colorectal tissue basally expresses high protein expression levels of Noxes^24^. In total, *Ca*.60% of CRC present a mutation on Ras proto-oncogenes or one of their downstream effectors^25^. Cells bearing Ras mutations exhibit enhanced ROS generation^26^. Nox1 expression correlated with KRas-activating mutations in CRC^27^. Ras proteins are GTPases that function as molecular switches regulating cell proliferation and survival through canonical Raf-MEK-Erk and PI3K pathways. Aberrant Ras overactivation often follows single mutations favoring GTP binding, typically at codons 12, 13, or 61 and associates with hyper-proliferative developmental disorders and cancer. Here, we investigated mechanisms associated with PDIA1-mediated Nox1 regulation in CRC exhibiting distinct levels of Ras pathway activation.

## Results

### Cell models

**Fig. 1:**
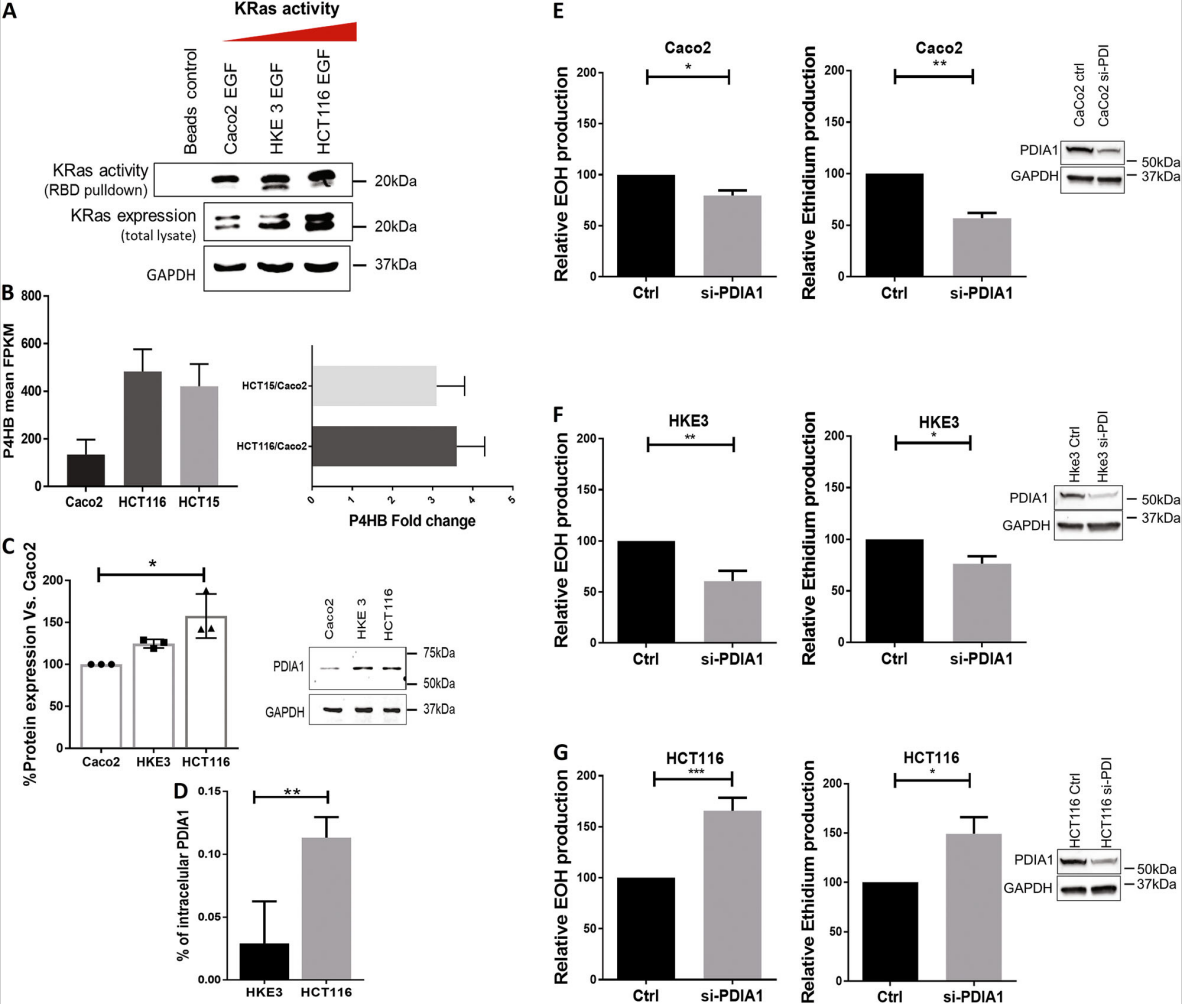
Role of PDIA1 in oxidant generation by colon carcinoma cells with distinct levels of KRas activation. **a** KRas expression and activity in Caco2, HKE3, and HCT116 cells. Active KRas was pulled down with GST-RBD beads from lysates of serum-starved cells treated with EGF 25 ng/mL for 10 min. Aliquots of the lysates were blotted for total KRas and GAPDH as loading control. **b** RNAseq analysis of P4HB (PDIA1) gene in Caco2, HCT116, and HCT15 cells, showing mean expression in FPKM (Fragments Per Kilobase Million), P4HB fold-change expression in HCT15 vs. Caco2 (q < 0.000001) and HCT116 vs. Caco2 (q < 0.000001). **c** PDIA1 basal protein expression by western analysis, normalized for GAPDH (*n* = 3) and quantified using Odyssey software. **d** Intracellular PDIA1 titration, using Human P4HB ELISA Pair Set (SinoBiological). Test *t* **p < 0.01, (*n* = 4); **e–g** ROS production after 72 h of PDIA1 silencing, measured by HPLC analysis of DHE oxidation products. DHE oxidation produces, among many others, two major products: 2-hidroxyethidium (EOH), which is representative of superoxide species and ethidium, representative of other oxidant species. Ctrl negative, si-RNA control; si-PDI, si-RNA against PDIA1 protein. Representative immunoblots of PDI silencing for each cell type Test *t* *p < 0.05; **p < 0.01. For Caco2 cells, *n* = 3; HKE3 cells, *n* = 4; HCT116 cells, *n* = 5

To assess the roles of PDIA1-dependent Nox regulation in CRC, we selected colon carcinoma cell lines HCT116 and HKE3, a well-known pair of isogenic cell lines known to differ by KRas constitutive activation (Fig. 1a and Suppl. Fig.1A). KRas^G13D^ mutant gene is disrupted in HKE3 cells by homologous recombination and insertion of a non-transcribed KRas^G12C^ mutant^28^. Although recent data^29^ showed that HKE3 cells do present a reminiscent RNA expression of KRas^G13D^ gene, KRas gene and protein are clearly less expressed in HKE3 than HCT116^29^. We also used a second control Caco2 colon carcinoma cell line, which is a non-mutated wild-type KRas control.

### PDIA1 expression correlates with Ras expression/activation

To further address whether KRas^G13D^ mutation correlates with PDIA1 expression, we performed RNAseq analysis comparing CRC cell lines presenting KRas^G13D^ mutation (HCT116, HCT15) vs. non-mutated KRas (Caco2) (Table 1). Our analysis showed that HCT116 and HCT15 cells display PDIA1 mRNA levels 3.6 and 3.1-fold, respectively, vs. Caco2 (Fig. 1b).

**Table 1:** Colon carcinoma cell line characterization and respective mutations

Cell line	Derived from	Stage	APC	BRAF	CDKN2A	CTNNB1	PK3CA	SMAD4	KRAS	TP53	References
*Caco2*	Primary tumor 72-year-old individual	ND	p.G1367*	wt	wt	p.G245A	wt	wt	wt	p.E204X	Fogh et al.^73^ Ilyas et al.^74^ Vijaya Chandra et al.^75^
*HCT15*	Primary tumor Male DLD1 isogenic cell	Dukes’C	p.I1417fs*2	wt	wt	wt	p.E545K p.D549N	wt	p.G13D	p.S241F	Dexter et al.^76^
*HCT116*	Primary tumor 48-year-old male	Dukes’D	Wt	wt	p.R24fs*20	p.S45del	p.H1047R	wt	p.G13D	wt	Brattain et al.^77^
*HKE3*	HCT116 isogenic cell	Dukes’D	Wt	wt	p.R24fs*20	p.S45del	p.H1047R	wt	p.G13D p.G12C	wt	Shirasawa et al.^28^ Fasterius et al.^29^
*HT29-D4*	HT29 clone Primary tumor 44-year-old female	Dukes’C	p.E853*	p.V600E	wt	wt	p.P449T	p.Q311*	wt	p.R273H	Fogh and Trempe^78^ Fantini et al.^79^

Focus on gene of **APC** adenomatous polyposis coli, **BRAF**, **CDKN2A** cyclin-dependent kinase Inhibitor 2A, **CTNNB1** catenin beta-1, **PK3CA** phosphatidylinositol-4,5-bisphosphate 3-kinase catalytic subunit alpha, **SMAD4** SMAD family member 4, **KRAS** Kirsten rat sarcoma viral oncogene homolog, **TP53**. All data were obtained from ATCC website plus above references. *stop codon; fs*: frame shift; del: deletion

In addition to bioinformatic analysis, we performed immunoblots to validate these results in our cells, showing increased PDIA1 protein expression in HCT116 vs. HKE3 or Caco2 (Fig. 1c), further confirmed through intracellular PDIA1 titration using ELISA (Fig. 1d). ER stress marker expression showed no differences among the cell lines (Suppl. Fig. 1B). As a control, we transfected primary VSMC with overactivated Ras mutant, and observed analogous increase in PDIA1 gene expression (Suppl. Fig. 1C). Thus, PDIA1 protein expression correlates with increased KRas activation.

### PDIA1 silencing promotes dual, Ras-dependent, effects on superoxide production

To address the effects of PDIA1 on oxidant generation, we investigated the effects of PDIA1 loss-of-function. Specific superoxide generation was assessed through DHE/HPLC method^30^. There was no difference in baseline superoxide generation between HKE3 and HCT116 cells (Suppl. Fig. 1D). We then examined the effects of PDIA1 loss-of-function through siRNA. Importantly, drastic PDIA1 knockdown is reportedly cytotoxic^31^ and unpublished results from our laboratory), and in vivo PDIA1 knockout is lethal for mice embryos^32^. Furthermore, PDIA1 protein is quite stable, with a long half-life^33^, requiring aggressive procedures to promote its complete silencing. Thus, here we performed a conservative loss-of-function using siRNA-mediated silencing, with 54-60% lower PDIA1 expression in our cells (Suppl. Fig. 2A). PDIA1 silencing promoted lower superoxide generation in HKE3 and Caco2 cells (Fig. 1e, f), in line with our previous VSMC data^21^. Contrarily, in HCT116 cells, PDIA1 silencing increased superoxide production (Fig. 1g). These results were confirmed using the lucigenin reductase assay (Suppl. Fig. 3). Thus, overactivated Ras associates with switch in the pattern of PDIA1-mediated superoxide regulation. We interrogated whether ER stress would be differentially induced by PDIA1 silencing in these cells. However, ER stress marker expression was unaltered by PDIA1 silencing in HKE3 and HCT116 cells (Suppl. Fig. 2B). Analogous effects occurred in HT29-D4 cells bearing V600E activating mutation on Ras effector Braf; PDIA1 silencing in these cells promoted increased superoxide production (Suppl. Fig. 3D). To further address whether overactivated Ras associates with switch in the pattern of PDIA1-mediated superoxide regulation, Caco2 cells were transfected with KRas^G13D^ active form, which promoted significant increase of superoxide production (Suppl. Fig. 4A). It is well known that in Caco2 cells, Ras activation induces Nox1 expression and increases ROS production through the MEK-ERK pathway and GATA6 transcription factor^34^. Opposite to wild-type Caco2 cells, in which PDIA1 silencing diminished superoxide production (Fig. 1e), transfection of the KRas^G13D^ active form blocked such effect (Suppl. Fig. 4B); however, this experiment proved to be technically difficult due to simultaneous transfections of KRas plasmid and PDIA1 siRNA oligonucleotide. Thus, we assessed the effects of thiomuscimol (15 µM, 15 min), a thiol inhibitor acting as a non-specific but cell-permeable PDIA1 inhibitor^35^. Thiomuscimol lead to increase in superoxide production, similar to that observed in HCT116 cells after PDIA1 silencing (Suppl. Fig. 4C, D).

### PDIA1 silencing sustains superoxide production in HCT116 through Nox1 NADPH oxidase

**Fig. 2:**
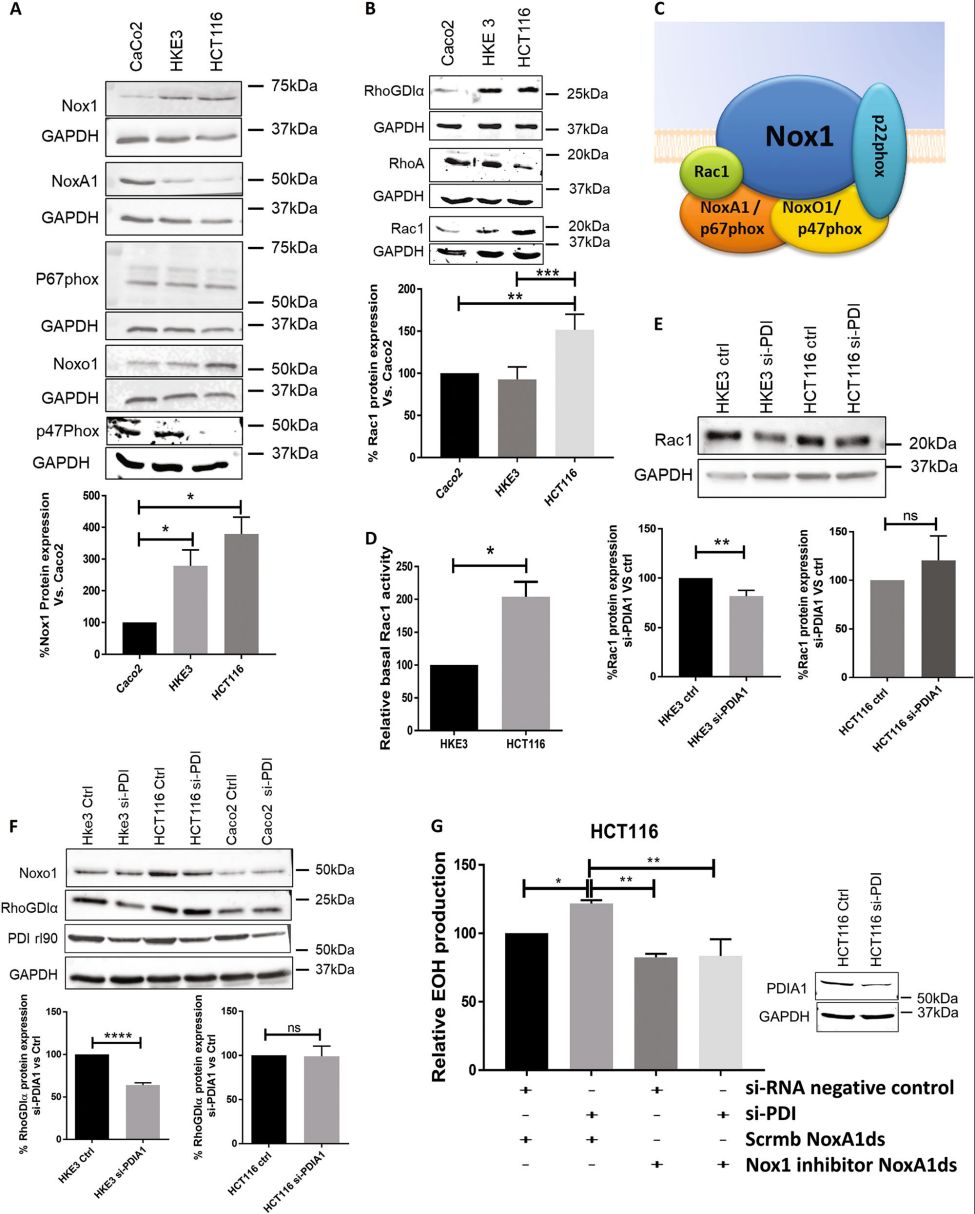
Nox NADPH oxidase expression and superoxide generation by HCT116 cells with overactivated KRas. **a** Nox1, NoxA1, p67phox, NoxO1, and p47phox basal protein expression by western analysis normalized for GADPH. Nox1 immunoblot densities were quantified using Odyssey software. *P < 0.05, ANOVA plus Tukey's multiple comparisons test (*n* = 3). **b** RhoGDIα, RhoA, and Rac1 basal protein expression by western analysis normalized for GAPDH. Rac1 immunoblot densities were quantified using Odyssey software. **P < 0.01; ***P < 0.005, ANOVA plus Tukey's multiple comparisons test (*n* = 3). **c** Nox1 complex scheme. **d** Rac1 basal activity, using Rac1 G-LISA activation assay (Cytoskeleton,inc). Test *t* *p < 0.05, (*n* = 2). **e** Rac1 protein expression by western analysis after 72 h of PDIA1 silencing. Rac1 Immunoblot densities were quantified using Odyssey software **P < 0.01; ns: not significant, Student's *t*-test (*n* = 3). **f** Western analysis of NoxO1 and RhoGDIα protein expression before and after PDIA1 silencing, normalized for GAPDH. RhoGDIα immunoblot densities were quantified using Odyssey software, ****P < 0.001; ns: not significant, Student's *t*-test (*n* = 3). **g** Superoxide production after 72h of PDIA1 silencing in HCT116 cells treated or not with 10 µM of NoxA1ds Nox1’s peptide inhibitor. EOH 2-hidroxyethidiium relative superoxide production, si-PDI, si-RNA against PDIA1 protein; Scrmb NoxA1ds, NoxA1ds negative control peptide; NoxA1ds, Nox1 peptide inhibitor. *P < 0.05; **P < 0.01, ANOVA plus Tukey's multiple comparisons test (*n* = 3)

To investigate the source of superoxide in our cells, we first assessed the protein expression of Nox complex subunits. Immunoblot analysis showed that Nox1 was more expressed in HKE3 and HCT116 vs. Caco2 control, consistent with the previously described changes in Nox1 due to Ras activation^25^ (Fig. 2a). Concerning regulatory Nox subunits (Fig. 2a, c), Nox organizer 1 (NoxO1, a p47phox analog) expression was significantly higher in HCT116 vs. other cell lines, while p47phox was more expressed in HKE3 and Caco2 (Fig. 2a). Caco2 cells expressed significantly more NoxA1 (p67phox analog Nox activator-1) than HCT116 and HKE3; however, p67phox expression was similar among these cells. Expression of RhoGTPases and their regulators was distinct among cell types: HCT116 cells expressed more Rac1 than Caco2 and HKE3, while RhoA expression showed an inverse pattern, with low expression in HCT116 cells. Meanwhile, Caco2 expressed less RhoGDIα than HKE3 and HCT116 (Fig. 2b). Rac1 activity was higher in HCT116 cells vs. HKE3 (Fig. 2d).

To investigate possible roles of Nox1 in oxidant generation in HCT116 cells, we used the Nox1 peptide inhibitor NoxA1ds^36^. Incubation with this cell-permeable peptide led to decreased superoxide production in HCT116, which was statistically significant after PDIA1 silencing (Fig. 2g). Therefore, Nox1 complex contributes to superoxide production in HCT116 cells. These data are in line with results in HT29-D4 cells, in which superoxide increase after PDIA1 silencing was prevented by concomitant Nox1 silencing (Suppl. Fig. 3D). We next addressed whether PDIA1 silencing affected protein expression of Nox1 or its regulatory subunits. PDIA1 silencing in HKE3 and HCT116 cells did not alter NoxO1 protein expression (Fig. 2f). Rac1 protein expression was slightly decreased by PDIA1 silencing in HKE3, but not HCT116 cells (Fig. 2e). Interestingly, PDIA1 silencing promoted decrease in RhoGDIα expression in HKE3 but not in HCT116 cells (Fig. 2f), in line with the Rac1 results. We propose that PDIA1 sustains Nox1 activity through RhoGDIα and Rac1, while in a context of KRas overactivation, KRas would bypass PDIA1/Nox1 regulation by directly sustaining high Rac1 activity in HCT116 cells.

### KRas overactivation bypasses PDIA1/Nox1 regulation by sustaining high Rac1 activity

**Fig. 3:**
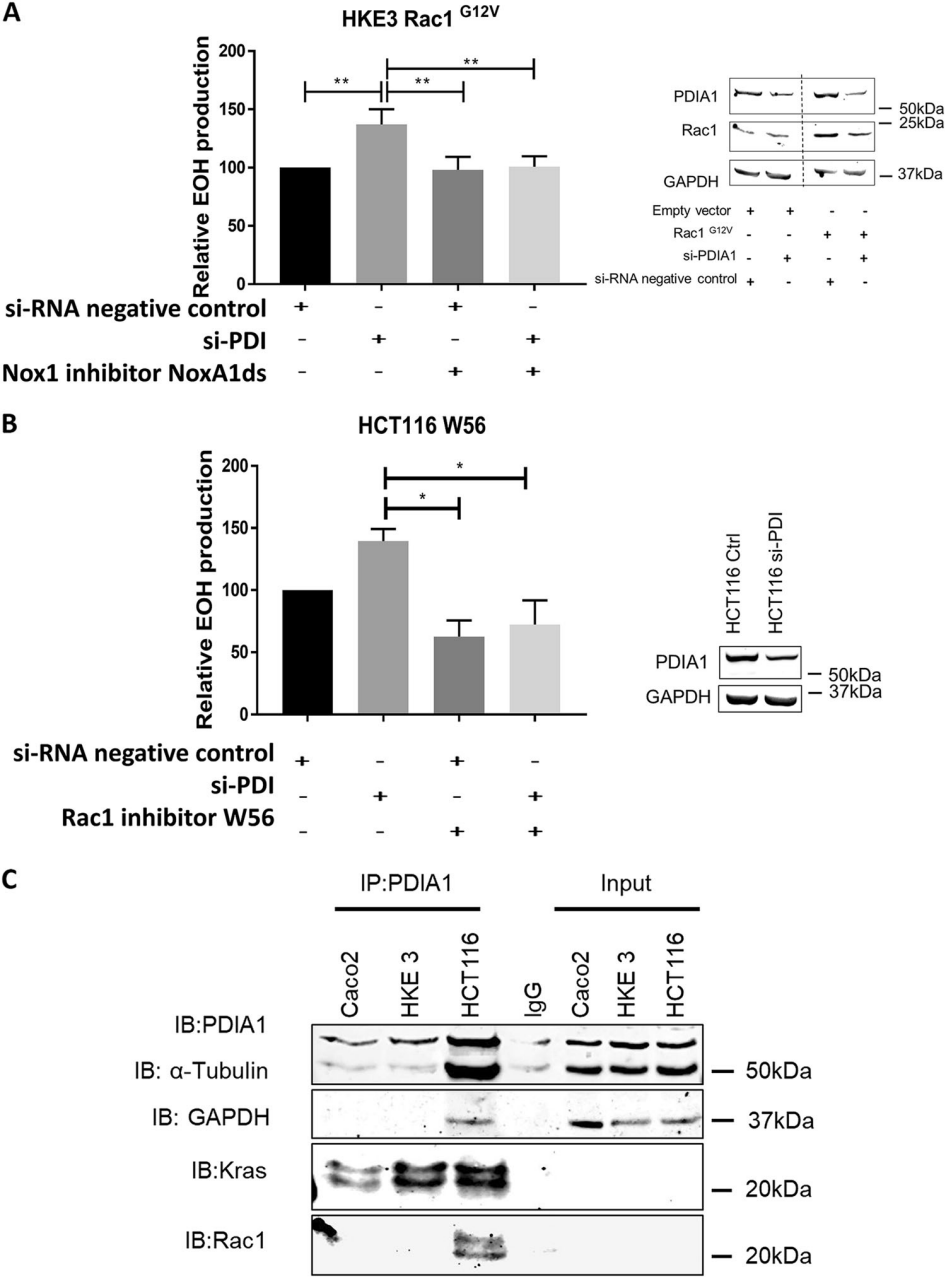
Rac1 and regulation of PDIA1 – Nox1 axis. **a** Superoxide production after 72 h of PDIA1 silencing in HKE3 transfected with Rac1^G12V^, treated or not with 10 µM of NoxA1ds (Nox1 peptide inhibitor), measured by DHE oxidation detected by HPLC. **p < 0.01, ANOVA plus Tukey's multiple comparison test (*n* = 3). **b** Superoxide production after 72 h of PDIA1 silencing in HCT116 cells treated or not with 50 µM of W56 Rac1 peptide inhibitor. *p < 0.05, ANOVA plus Tukey's multiple comparison test (*n* = 4). **c** PDIA1 co-immunoprecipitation, IP: PDIA1 immunoprecipitation in Caco2, HKE3, and HCT116 cells, IgG: Immunoglobulin control, Input: 1% of total protein lysate, IB: immunoblot against PDIA1, KRas, Rac1, GAPDH, and tubulin (*n* = 2). EOH: 2-hidroxyethidiium relative superoxide production

To investigate whether Rac1 activation could account for the sustained Nox1 activation in HCT116 cells, HKE3 cells were transfected with overactive Rac1^G12V^ mutant in a way to mimic HCT116 cells. Contrarily to non-transfected HKE3 cells, HKE3 Rac1^G12V^ showed increased superoxide production 72 h after PDIA1 silencing (Fig. 3a), similar to results from HCT116 cells (Fig. 1g). Conversely, treatment of HCT116 cells with the Rac1 peptide inhibitor W56^37^ decreased basal superoxide levels and prevented superoxide increase after PDIA1 silencing (Fig. 3b). These results suggest that constitutive high Rac1 activation may contribute to the switch of PDI-dependent regulation from supporting to limiting superoxide production, respectively, in cells with low levels vs. overactivated KRas.

### PDIA1 interaction with KRas and Rac1

The results so far allow the proposal that PDIA1 may act as a servomechanism (oscillator) basally supporting, while in parallel posing a "capping" limit to superoxide generation, depending on the enhanced activations of Rac1 and KRas. We investigated possible interactions between PDIA1 and KRas. Using cavitation techniques (see Methods) able to preserve sensitive protein complexes and/or microdomains, given that KRas is known to localize to specific labile nanoclusters^38^, we showed that PDIA1 coimmunoprecipitated with KRas (Fig. 3c) in all cell types, with enhanced detectable interaction in HCT116. PDIA1 immunoprecipitation yielded enhanced protein amounts in HCT116 vs. HKE3 cells, consistent with results from Fig. 1c, d. Moreover, PDIA1 also exhibited detectable interaction with Rac1 (Fig. 3c). Similar interactions between PDIA1/KRas and PDIA1/Rac1 were also detected in HUVEC (Suppl. Fig. 5), while we have previously detected PDIA1-Rac1 interaction in VSMC^21^.

### Functional effects of PDIA1 silencing on cell proliferation and migration

**Fig. 4:**
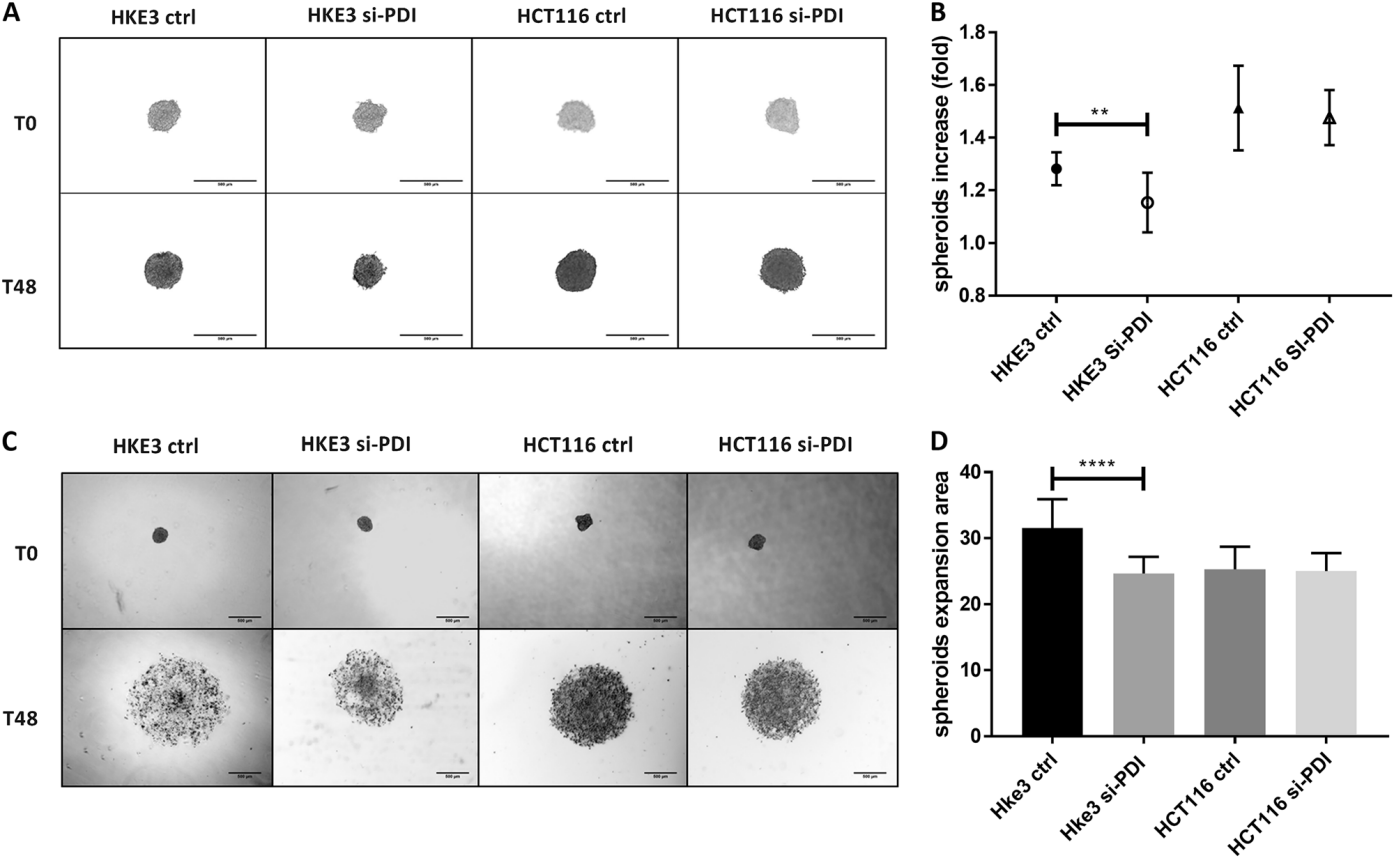
Effects of PDIA1 silencing on spheroid growth and invasiveness. 3D cell proliferation assay: **a** Representative phase-contrast images of spheroid proliferation with or without PDIA1 silencing; pictures were taken at T0 and T48 h after spheroid formation on methylcellulose media. Scale bar, 500 µm. **b** Proliferation analysis: spheroid surface area was measured at T0 and T48 h, using ImageJ software. Spheroid growth was calculated as *T48 h spheroid area/T0 spheroid area*; between 17 and 27 spheroids were analyzed for each condition. Data are mean fold ± SD, **p < 0.01; ****p < 0.0001 vs. HKE3 scrmb, ANOVA plus Tukey's multiple comparison test. **c** Effect of PDIA1 silencing on cell invasion: representative phase-contrast images of spheroid invasion in 2D fibronectin matrix (10 µM); pictures were taken at T0 and T48 h after spheroids were laid down on matrix. Scale bar, 500 µm. **d** Spheroid 2D invasion analysis: total spheroid expansion was measured at T0 and T48 h using ImageJ software. Spheroid expansion was calculated as *(T48 h total evasion area—T0 initial spheroid area)/T0 initial spheroid area*; between 12 and 17 spheroids were analyzed for each condition. Data are mean ± SD, ****p < 0.0001 vs. HKE3 scrmb, ANOVA plus Tukey's multiple comparison test

Since ROS closely correlate with cell proliferation, migration, and survival^39,40^, we sought to investigate functional readouts of PDIA1 effects in tumor dynamics. The spheroid assay, also termed 3D culture, can provide information of cell proliferation and evasion, mimicking cell interaction, and escape from the core tumor. The growth of tumor cell spheroids was investigated by measuring total spheroid area at T0 and T48 h, calculated as the ratio *T48h spheroid area/T0 initial spheroid area*. HCT116 growth was expectedly higher vs. HKE3 cells. PDIA1 silencing induced decreased spheroid growth for HKE3, but not for HCT116 cells (Fig. 4a, b). To assess cell evasion after spheroid formation, spheroids were placed on fibronectin 2D matrix and their areas assessed at T0 and T48 h. Cell evasion was calculated as *(T48h total evasion area—T0 initial spheroid area)/T0 initial spheroid area*. PDIA1 silencing promoted decrease in cell evasion in HKE3 and no modification in HCT116 (Fig. 4c, d). These data are consistent with results of a 2D random migration assay in HT29-D4 cells (Suppl. Table 1) and are in line with previous data in VSMC^21^.

### Screening of cell signaling routes affected by PDIA1 silencing highlight GSK3β and Stat3

**Fig. 5:**
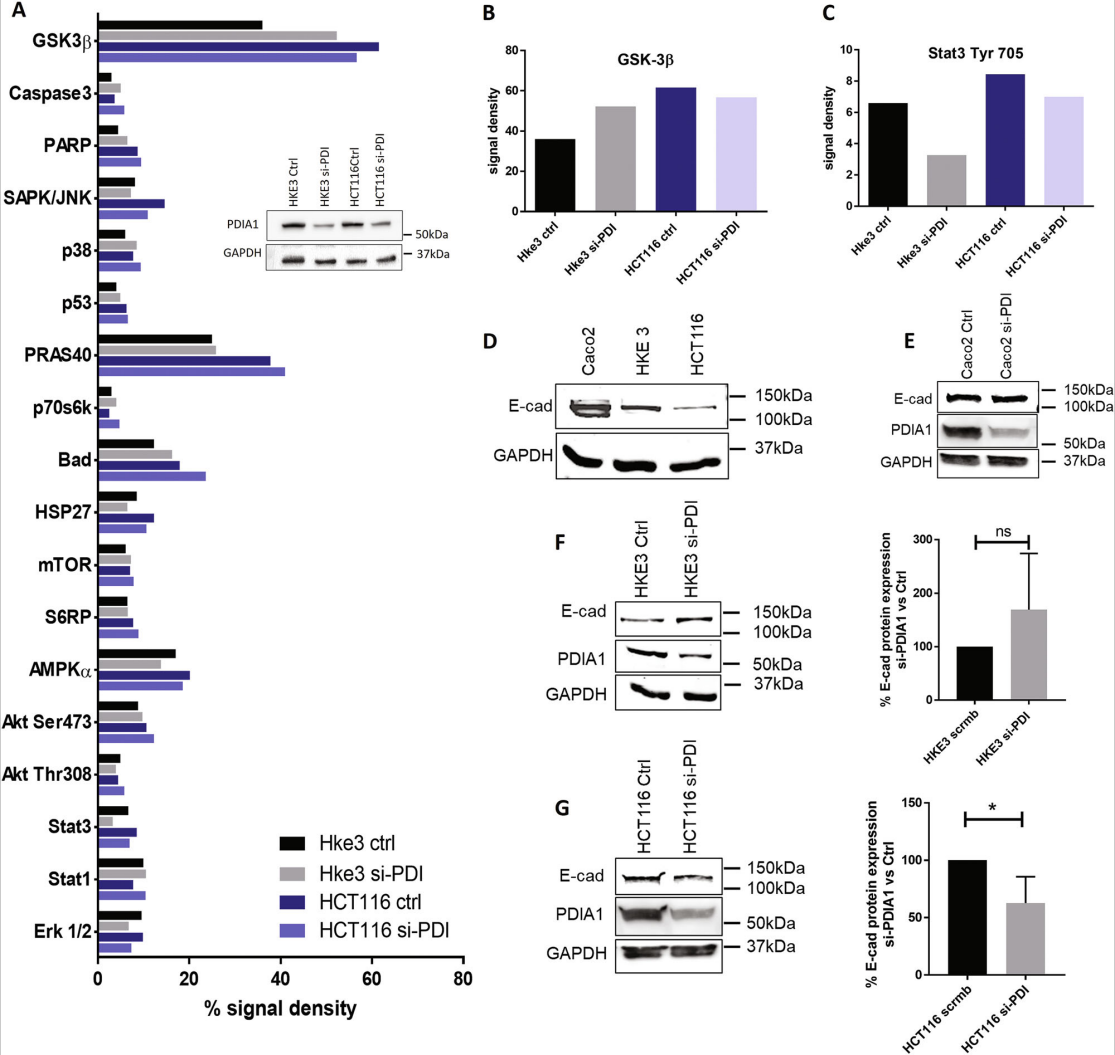
Effects of PDIA1 silencing in signaling targets and EMT marker expression. PathScan array analysis. All array’s spots were quantified and analyzed using ImageJ software. PDIA1 silencing was checked by immunoblot analysis. Ctrl, si-RNA negative control; si-PDI, si-RNA against PDIA1; ERK1/2, Thr202/Tyr204 phosphorylation; Stat1, Tyr701 phosphorylation; Stat3, Tyr705 phosphorylation; Akt, Thr308 phosphorylation; Akt, Ser473 phosphorylation; AMPKa, Thr172 phosphorylation, s6 ribosomal protein, Ser235/236 phosphorylation; mTOR, Ser2448 phosphorylation; HSP27, Ser78 phosphorylation; Bad, Ser112 phosphorylation; p70 S6 kinase, Thr389 phosphorylation; PRAS40, Thr246 phosphorylation; p53, Ser15 phosphorylation; p38, Thr180/Tyr182 phosphorylation; SAPK/JNK, Thr183/Tyr185 phosphorylation; PARP, Asp214 cleavage; Caspase-3, Asp175 Cleavage; GSK-3β, Ser9 phosphorylation. **b** GSK-3β, Ser9 phosphorylation plot. **c** Stat3, Tyr705 phosphorylation. **d** Basal E-cadherin (E-cad) protein expression by immunoblot normalized for GAPDH (*n* = 3). **e** Immunoblot for E-cadherin before and after PDIA1 silencing in Caco2 cells (*n* = 2). **f** Immunoblot for E-cadherin before and after PDIA1 silencing in HKE3 cells.; ns: not significant, Student's *t* test (*n* = 3). **g** Immunoblot for E-cadherin before and after PDIA1 silencing in HCT116 cells *P < 0.05, Student's *t* test (*n* = 3). Immunoblot densities were quantified using Odyssey software

Having shown a role for sustained Rac1 activation and different effects of PDIA1 silencing in cell evasion and proliferation, we further addressed potential signaling mechanisms underlying disrupted PDIA1-mediated superoxide regulation in CRC with Ras overactivation. For that, we screened major cell signaling pathways using PathScan^®^ Intracellular Signaling Array Kit, which is based on sandwich immunoassay principle, showing activation state of 18 key cell signaling proteins by their specific phosphorylation or cleavage. The assay was performed in HKE3 and HCT116 cells after PDIA1 silencing (Fig. 5a). We identified nine protein target phosphorylation or cleavage enhanced in HCT116 vs. HKE3: Stat3, GSK3β, p70, S6-ribosomal protein, HSP27, Bad, PARP, p38, Caspase-3, and PRAS40. Except for the latter, each of these was affected to some extent by PDIA1 silencing (Suppl. Fig. 6).

Given the slight increases in PARP cleavage and Caspase-3 phosphorylation after PDIA1 silencing, we performed a flow cytometry analysis of propidium iodide/annexinV labeling (Suppl. Fig. 7) and showed no significant difference between PDIA1-silenced cells vs. controls. Thus, the lower growth rates of HKE3 cells after PDIA1 silencing vs. baseline in the spheroid assay were not due to cell death.

Among the investigated pathways, Stat3 and GSK3β showed the most consistent results and were differentially affected by PDIA1 silencing between HKE3 and HCT116 (Fig. 5b, c). GSK3β (Glycogen synthase kinase-3 beta) is a constitutively active protein kinase inactivated by Ser9 phosphorylation. GSK3β supports Stat3 activation^41^. Stat3 is a transcription factor for many cytokines and growth factor receptors, activated by Tyr705 phosphorylation, which induces its dimerization, nuclear translocation, and DNA binding. In HKE3 cells, PDIA1 silencing induced Ser9 GSK3β phosphorylation (suggestive of inactivation) and reduced Tyr705 Stat3 phosphorylation (suggesting impaired activation) upon PDIA1 silencing. Conversely, in HCT116 cells, Ser9 phosphorylation of GSK3β was significantly elevated already at baseline and stayed elevated upon PDIA1 silencing. Meanwhile, Stat3 Tyr705 phosphorylation was also enhanced at baseline and remained high after PDIA1 silencing. Results with GSK3β were validated by immunoblot (Suppl. Fig. 8). These results indicate that GSK3β/Stat3 regulation is disrupted in HCT116 cells. Since Stat3 is a well-known target of Rac1^42^, we propose this may be a possible mechanism to sustain Stat3 activation in HCT116 cells.

Both GSK3β and Stat3 support tumorigenesis and metastasis induction through processes that include epithelial–mesenchymal transition (EMT)^41^. A recent study in HCT116 cells suggested that Rac1 overexpression/overactivation promotes EMT through Stat3^43^. Moreover, Stat3 negatively regulates expression of the epithelial phenotype marker E-cadherin in CRC^44^. We investigated whether EMT could be a mechanism by which PDIA1 silencing promoted both decreased cell evasion and lower Stat3 activation in HKE3 vs. no change in HCT116 (Fig. 4; Fig. 5a–c), focusing on E-cadherin protein expression. HCT116 cells expressed less E-cadherin vs. HKE3 and Caco2 at baseline (Fig. 5d). In parallel, E-cadherin protein expression was further decreased by PDIA1 silencing in HCT116, indicating switch to a mesenchymal phenotype (Fig. 5g). These results are in line with the disrupted regulation of the GSK3β/Stat3 axis in HCT116 cells. E-cadherin protein expression was unaltered in Caco2, probably due to their APC mutation (Fig. 5e).

### Enrichment pathway analysis of protein interaction networks

**Fig. 6:**
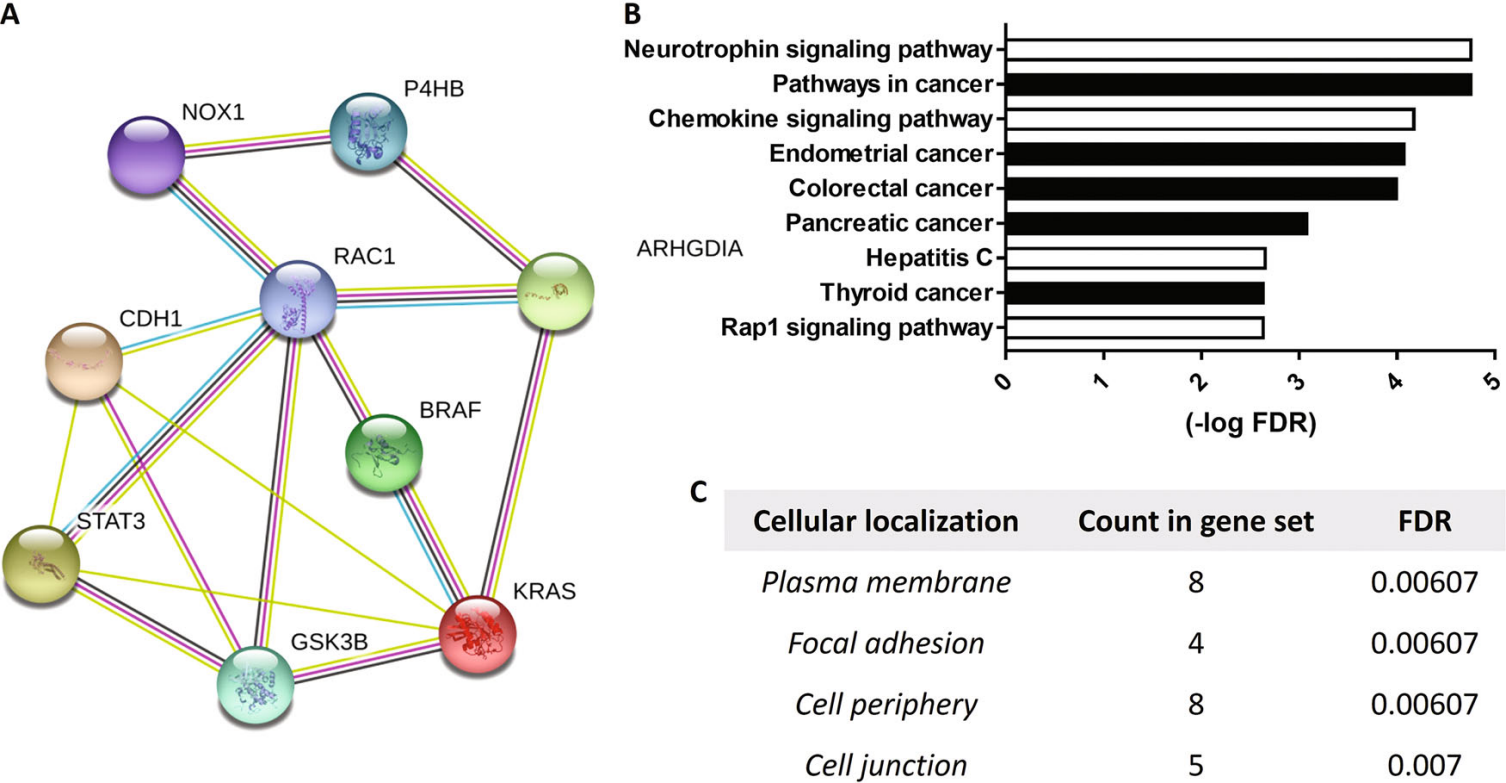
Analysis of protein–protein interaction network and functional pathways associated with PDIA1–Nox1 axis and KRas. **a** Interaction map fashioned with String 10.5 program (http://string-db.org), Network nodes represent proteins of Nox1, PDIA1 (P4HB), RhoGDIα (ARHGDIA), RAC1, BRAF, KRAS, GSK3β (GSK3B), STAT3, and E-cadherin (CDH1). Different colored lines displays predicted functional links. Interactions experimentally determined appear in pink, interactions curated from databases in blue, co-expression was represented in black and text data mining in green. The analysis showed: nine nodes with an average node degree of 3.56; number of edges: 16; expected number of edges: 4; average local clustering coefficient: 0.333; PPI enrichment *p*-value: 2.02e^−05^. **b** Top 10 ranked enriched proteins KEGG pathway analysis. Bar graphs show the –log of FDR (False discovery rate). High values correlated to higher probabilities. **c** Cellular component Gene ontology (GO) analysis

To contextualize our results, we constructed a protein–protein interaction network based upon enrichment pathway analysis, fashioned using string functional association network, in which nodes signify each protein and edges represent protein interactions. Interactions among Nox1, PDIA1, RhoGDIα, Rac1, B-Raf, KRas, GSK3β, STAT3, and E-cadherin were investigated and confronted to the database (Fig. 6a). The network connecting these nine proteins of interest associated with more edges (16) than those predicted by the software (4), suggesting that these proteins interact among themselves at a degree higher than that expected from an analogous stochastic group. Such an enrichment indicates that these proteins display some degree of biological connection as a group. Importantly, this analysis showed Rac1 as a likely hub of interaction with most proteins. Top 10 ranked enriched proteins by KEGG pathway analysis (Fig. 6b) showed five cancer-related pathways (black bars), including those for CRC. Gene Ontology (GO) analysis (Fig. 6c) identified proteins localized in the plasma membrane, focal adhesion, cell periphery, and cell junction.

## Discussion

Our results suggest the possibility that PDIA1, known to correlate with sustained tumor growth and metastatization^12,45^, may play significant regulatory roles in redox-related transition from less to more aggressive stages of tumorigenicity. Increased aggressiveness correlates with disabled PDIA1-mediated oxidant generation, resulting in PDIA1-supported restriction of oxidant generation. Whether this process is a possible component of a tumoral escape program deserves further discussion. ROS production can support either pro- or anti-tumoral effects, depending on the type, levels, and sources of ROS, as well as associated activated pathways. ROS production is a well-known hallmark of cancer initiation via DNA damage and genomic instability^46^. Redox signaling sustains crucial oncogenic processes, such as proliferation and migration^39,40^. On the other hand, high level of ROS can have anti-tumoral effects by inducing senescence and apoptosis^16^. Similarly, ROS generation interplays in different ways with responsiveness to anti-cancer drugs. Antioxidant supplementation shows no benefit and can even worsen cancer treatment in animal models^47,48^ and patients^49^. Efficacy of radiotherapy or some chemotherapeutics depends on their ability to induce ROS production. In HT29-D4 and Caco2 cells, Nox1 silencing significantly decreases oxaliplatin efficiency^50^, while adjuvant ROS-generating molecules are promising^51^. In contrast, sustained ROS production may associate with chemoresistance. CRC resistant to oxaliplatin present increased basal Nox1 activity and supportive of cell survival^52^.

Ras overactivation also presents a dual pattern of associated ROS-related effects. In Caco2 cells, Ras activation induces Nox1 expression through the MEK-ERK pathway and GATA6 transcription factor^34^. Ras overactivation-increased ROS production^26^ may associate with senescence and apoptosis^53,54^, but also with cancer initiation and sustained tumorigenicity^55^. In NRK cells (rat kidney fibroblast), cancer initiation by KRas^G12V^ requires Nox1, while KRas^G12V^-induced Nox1 upregulation requires Ras–Raf–MEK–ERK axis^56^. Therefore, Nox1 is important to maintain Ras-induced malignant transformation. Meanwhile, mitochondrial ROS also support Ras-dependant cancer cell transformation^57^. Mutant overactive KRas is also able to induce NRF2 upregulation, which buffers cellular oxidant levels but confers resistance to platine compounds^58,59^. The identification that the supportive vs. inhibitory patterns of PDIA1-dependent Nox-dependent regulation accompany Ras activation levels indicates that PDIA1 acts as an upstream determinant of redox signaling programs associated with distinct stages of tumorigenicity. Whether PDIA1-mediated servomechanism-like behavior at these distinct stages can modify tumor evolution remains to be determined.

Mechanisms involved in PDIA1-dependent superoxide modulation converge to RhoGTPases. We show that basal Rac1 levels closely correlate with the dual effects of PDIA1 on superoxide, since forced Rac1 overactivity in HKE3 induces PDIA1 to inhibit, while Rac1 inhibition in HCT116 induces PDIA1 to support Nox1-dependent superoxide. Since constitutive Rac1^G12V^ mutation likely bypasses its regulation by PDIA1, our experiments (Fig. 3) suggest that loss of Rac1 regulation by PDIA1 can be a key mechanism underlying transition from PDIA1-supported to PDIA1-inhibited ROS generation. In parallel, since Ras activity correlates with such PDIA1 effects, we speculate that Ras-mediated Rac1 activation underlies PDIA1 bypass and transition in oxidant regulation. PDIA1–Rac1 interaction accounts for PDIA1 effects on VSMC Nox1-mediated processes, as PDIA1 silencing disables the associated Rac1 activation and PDIA1 interacts with Rac1^21^. Here, we confirmed a similar PDIA1–Rac1 interaction both in CRC and endothelial cells. Moreover, PDIA1 has been associated with significant effects on cytoskeletal regulation, which is the hallmark of RhoGTPase effects^60,61^. The importance of PDI-RhoGTPase convergence is further evident from our recent study showing an extremely conserved evolutionary pattern of gene clustering involving PDI and RhoGDI families. Since RhoGDIs are essential regulators of RhoGTPase activity and PDIA1 closely interacts with RhoGDIα^62^, RhoGDIs may be a mechanism whereby PDIA1 regulate Rac1, as indeed suggested by our expression data (Fig. 2c–e). These considerations further support Rac1 as a crucial node at the center of a signaling hub involving PDIA1, RhoGDIs, Ras, and other proteins, as indeed shown in our protein–protein interaction model (Fig. 6).

In HCT116 and HT29-D4 cells, in which ROS production stays elevated after PDIA1 silencing, there is associated insensitivity of cell migration to PDIA1 silencing (Fig. 5). An important mechanism supporting cell migration is EMT, known to associate with ROS production in breast cancer cells^63^. In melanoma, Nox1-derived ROS sustains and Nox1 inhibition reverts EMT^64^. PDIA1 silencing promoted switch to epithelial phenotype in HKE3 cells, but a switch to mesenchymal phenotype in HCT116 cells. It is possible that PDIA1 supports EMT through its effects on Nox1 and ROS. Our results are in line with previous reports in hepatocellular carcinoma showing that PDIA1 supports tumorigenesis by enhancing EMT through Grp78 downregulation^65^. Furthermore, E-cadherin protein expression negatively correlates with Stat3 activation in CRC^66^. Indeed, in our HKE3 cells, PDIA1 silencing induces Stat3 inhibition and increased E-cadherin expression. Rac1 sustains Stat3 activation;^42^ we believe that Ras-induced sustained Rac1 underlies the lack of effect of PDIA1 silencing on Stat3 (Fig. 4). Basal saturation of these pathways may explain why HCT116 cells, despite exhibiting more mesenchymal phenotype after PDIA1 silencing, does not further enhance cell invasion. Of note, Stat3 activation has been correlated with Nox1 expression^67–69^. Overall, the GSK3β/Stat3 axis deserves further investigation regarding the pathways whereby PDIA1 interplays with Nox1 activation during Ras overactivation and EMT.

**Fig. 7:**
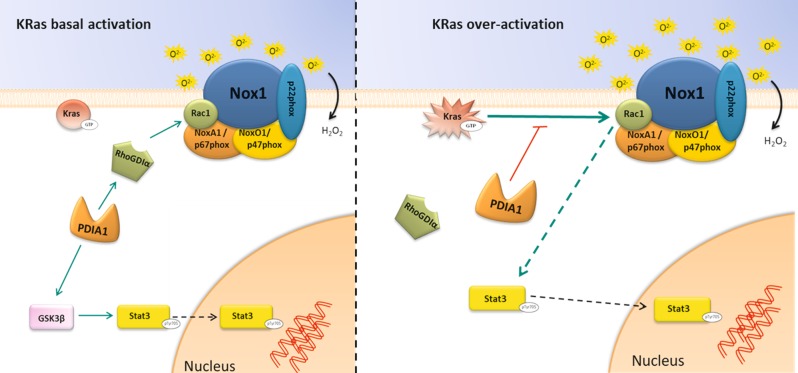
Model of PDIA1-associated regulation of Nox1 in cells with normal or overactivated KRas

Altogether, our results indicate (Fig. 7) a novel dual effect of PDIA1 on Nox1-dependent superoxide regulation in CRC, as a servomechanism correlating with the extent of Ras activation. Such Ras-induced bypass of the otherwise normal support of Nox1 by PDIA1 seemingly involves an independent activation of Rac1. Several studies indicate that primarily adaptive responses, e.g., senescence, can be hijacked to promote tumor escape responses, such as stemness^70^. PDIA1 could thus act as a regulatory mechanism underlying adaptive redox switches from a tumor-suppressive to a vicious adaptive program promoting tumor escape. These results reinforce the emerging potential therapeutic implications of PDIA1 inhibition against cancer progression.

## Methods

### Reagents

Unless otherwise stated, reagents were from Sigma. DPI (10 μM) was from Merck Millipore; dihydroethidium (DHE) molecular probes ref. D1168; W56 (Rac1 inhibitor) was from TOCRIS 2221. NoxA1ds (Nox1 inhibitor) was synthesized at the Department of Biophysics, UNIFESP, Sao Paulo, (as described in ref. ^36^) NoxA1ds peptide sequence NH_3_-EPVDALGKAKV-CONH_2_, Scrambled NoxA1ds peptide sequence NH_3_-LVKGPDAEKVA-CONH_2_ .

### Cell culture

Human colon carcinoma cell lines, HCT116 and HKE3 are a gentle donation from MD. Walter Kolch (University College Dublin, Belfield, Dublin 4, Ireland). HCT116 and HKE3 cells were maintained in Dulbecco’s modified Eagle’s medium (DMEM) supplemented with 10% fetal bovine serum (FBS) (GIBCO Cell Culture systems, Invitrogen), at 37 °C in a humidified atmosphere with 5% CO_2_. Caco2 cells were maintained in DMEM supplemented with 10% FBS and non-essential amino acids (from sigma, ref. M7145), at 37 °C in a humidified atmosphere with 5% CO_2_.

### Cell transfection

HKE3 and HCT116 and Caco2 were transiently transfected using Amaxa nucleofactor^TM^ system from Lonza, using Kit V (ref. VCA-1003) according to the manufacturer's protocols (Nb: HKE3 cells were transfected using the same protocol as HCT116). For PDIA1 silencing, cells were transfected with 300 nmol of PDIA1 triplex si-RNA (from OriGene) or stealth RNAi medium Universal negative control (from Invitrogen). All PDIA1 silencing experiments were performed after 72 h of silencing. Overexpression of constitutively active Rac1 was achieved by transfection of 2 µg pCDNA3.1-Rac1 G12V plasmids or 2 µg pCDNA3.1 empty vector; cells were analyzed 72 h after transfection.

### Immunoblot analysis

Equal amounts of protein from lysates were resolved by SDS-PAGE. The following primary antibodies were used: anti-GAPDH (1/20,000 ref. ab8245) anti-KRas (1/500 ref.137739); anti-Nox1 (1/1 000 ref. ab121009); anti-NoxA1 (1/1 000 ref. ab68523); anti-NoxO1 (1/1000 ref. ab34761); anti-Rac1 (1/1000 ref. ab33186); anti-RhoGDI (1/2000 ref. ab53850) from Abcam, anti-GSK3 total (1/1000 ref.5676); anti-pGSK3 (1/1000 ref. 9331) from cell signaling, anti-RhoA (1/1000 ref. SC26C4) from Santa Cruz Biotechnology, anti-GAPDH (1/20,000, ref. G8795) from Sigma, anti-KDEL (1/1000, SPA-827) from stressgen, anti-PDIA1 (1/1000, clone RL90 ref. MA3-019) from Thermo. The HRP-coupled secondary antibodies were purchased from Cell Signaling Technology (1/5000). Fluorescent-coupled secondary antibodies were purchased from Odyssey (1/10,000, anti-mouse ref. 926-32212, anti-rabbit 926-32223, anti-goat ref. 926-32224), and fluorescent immunoblotting were scanned with the Odyssey near-infrared imaging system.

### PDIA1 quantification by ELISA

In total, 2 x 10^6^ cells was seed in 100 mm cell culture dish, 24 h after seeding cell were lysed in 300 µL of lysis buffer A (HEPES 20 mM, NaCl 150 mM, Glycerol 10%, triton 1%, EGTA 1 mM, MgCl_2_ 1.5 mM). Following cell lysis, soluble PDIA1 antigen was measured using the Human P4HB Pair Set enzyme-linked immunosorbent assay (ELISA) (SinoBiological Inc) according to the protocol described by the supplier. Briefly, 96-well microplate was coated with 100 µl per well of the diluted capture antibody and incubated overnight at 4 °C. Thereafter, each sample was added for 2 h at room temperature. Following 100 µl of detection antibody conjugated to horseradish-peroxidase (HRP) was incubated for 2 h at room temperature. Plates were washed three times after each incubation. Finally, 200 µl of tetramethylbenzidine (TMB) solution were added for 30 min, and optical density of each well were determined immediately using a microplate reader (SpectraMax 340, Molecular Devices) set to 450 nm. The values were determined according to a standard curve, and normalized according to the total protein dosage.

### Measurement of Rac1 and Ras activity

Rac1 activity was assessed by G-lisa ref.BK128 protocol, as furnished by the manufacturer Cytoskeleton cells were starved 16 h before assay. For the pulldown assay of activated KRas, cells were starved for 16 h and incubated for 10 min with 25 ng/mL of EGF. Cells were harvested on G-lisa lysis buffer (ref. GL36 from cytoskeleton) and centrifuged at 12,000*g*, 10 min, 4 °C. Homogenates (600 µg) were incubated for 2 h with glutathione S-transferase-Ras binding domain (GST-RBD, to detect active KRas) previously coupled to glutathione–Sepharose (ref. 17-0756-01 from GE Health Care) at 4 °C under gentle agitation. After washing three times (50 mM Tris, pH 7.5, 0.5% Triton X-100, 150 mM NaCl, 5 mM MgCl_2_), proteins retained on the resins were released in Laemmli sample buffer and boiled for 5 min at 100 °C. Proteins were analyzed by immunoblotting with anti-KRas antibody.

### Coimmunoprecipitation Experiments

Cells 10 × 10^6^ were grown to confluence in 150 mm plates, washed three times in PBS buffer and 2 ml of lysis buffer (20 mM Tris-HCl pH 7.8, 250 mM sucrose, 1 mM MgCl_2_, and 1 mM CaCl_2_) supplemented with protease (1 mM PMSF, 1 µg/ml leupeptin and aprotinin) and phosphatase (50 mM sodium fluoride, 2 mM sodium orthovanadate, 10 mM sodium pyrophosphate) inhibitors. Cells kept for 20 min over ice were scraped and collected in a final volume of 5 ml. The cell suspension was transferred to a 35 -ml nitrogen cavitation bomb for 30 min in 400-psi nitrogen pressure on ice. Intact cells, large debris and nucleus were removed by centrifugation at 1 000 g for 10 min at 4°C. Lysates were incubated overnight at 4 °C under agitation with 8 µg of PDIA1 antibody, followed by incubation with 70 μl of protein G-coated magnetic beads (ref. 28-9513-79 from GE Health Care) for 4 h at 4 °C. Beads were successively washed in sucrose buffer to remove contaminating material, ressuspended in modified FLAG lysis buffer (50 mM Tris-HCl pH 7.4, 150 mM NaCl, 1 mM EDTA, 1% Triton X-100 and 1% CHAPS) supplemented with a protease and phosphatase inhibitors. After 1 h of incubation at room temperature, Laemmli sample buffer was added and incubated at room temperature for additional 1 h. KRas was detected by immunoblot.

### Detection of ROS production

Intracellular cell ROS production was assessed by HPLC analysis of dihydroethidium (DHE)-derived oxidation products, as described^30^. DHE oxidation produces, among many others, two major products: 2-hydroxyethidium (EOH), which is representative of superoxide species, and ethidium, representative of other oxidant species. Cells were starved for 4 h and incubated or not 2 h with 10 µM of the Nox1 peptide inhibitor NoxA1ds or 50 µM of W56 Rac1 peptide inhibitor. Cells were washed with HBSS without phenol red, Ca^2+^, and Mg^2+^, and incubated for 30 min with 100 μM DHE, plus the inhibitors, on HBSS without Ca^2+^ and Mg^2+^. Cells were washed with cold PBS, harvested in 500 µl acetonitrile and centrifuged (12,000 × *g* for 10 min at 4 °C). The homogenate was dried under vacuum and analyzed by HPLC with fluorescence detectors (Waters 2475 HPLC, Colum Synergi 4µ Polar-RP 80A from Allcrom ref. 00F-4336-E0). Quantification of DHE, EOH, and ethidium concentrations was performed by comparison of integrated peak areas between the obtained and standard curves of each product under identical chromatographic conditions. EOH and ethidium were monitored by fluorescence detection with excitation 480 nm and emission 580 nm, whereas DHE was monitored by ultraviolet absorption at 245 nm. Results were expressed as calculated EOH or ethidium concentrations (micromolar), normalized for consumed DHE (i.e., initial minus remaining DHE concentration in the sample).

### PathScan^®^ intracellular signaling array

Signaling pathways associated with the effects of PDIA1 loss-of-function were investigated by the PathScan^®^ Intracellular Signaling Array Kit (ref. 7323), which is a slide-based antibody array optimizing the performance of sandwich immunoassays. The kit allows the simultaneous detection of 18 signaling molecules (described in Fig. 5), which are either phosphorylated or cleaved. Target-specific capture antibodies were spotted in duplicate onto nitrocellulose-coated glass slides. Quantification of all spot intensities was performed using ImageJ software.

### Cell migration assays spheroids

Three-dimensional cell invasion assay was adapted from previously published works^71,72^. Twenty-four hours after cell transfection with PDIA1, si-RNA cells are trypsinized, counted, and re-suspended in complete medium containing 2.4 mg/ml methylcellulose. In order to start the experiment with equivalent-size T0 spheroids, 1,500 cells per well were seeded for HKE3 vs. 1000 for HCT116. The suspension (100 µl) was added into each well of a U-bottom 96-well plate, allowing the formation of one spheroid per well. Twenty-four hours after plating (T0), spheroids were transferred to a flat-bottom 24-well plate coated with 10 µg/mL fibronectin. Pictures were taken at T0 and T48 h in a Olympus microscope, objective 2X. Invasion was quantified by measuring the area occupied by cells spheroid expansion at T0 and T48 h using ImageJ software. Spheroid expansion was calculated as: (T48 h total evasion area—T0 initial spheroid area)/T0 initial spheroid area. For the proliferation analysis, 24 h after plating (T0), spheroids stayed on a U-bottom 96-well plate with methylcellulose media, and spheroid surface areas were measured at T0 and T48 h using ImageJ software. Spheroid growth was calculated as: T48 h spheroid area/T0 spheroid area. T0 spheroids out of mean size range were excluded from the analysis.

### RNASeq data mining

Experiments used: Caco2: SRR1580950, SRR4249634, SRR4249633, SRR4249636, SRR1581012, SRR4249635. HCT116: SRR1636085, SRR3228429, SRR5009474, SRR5297165, SRR1636086, SRR3228430, SRR5009521, SRR5297166, SRR1636087, SRR5009406, SRR5009538, SRR902610. HCT15: SRR1756568, DRR046626, and ERR208903.

Experiments of the lineages listed were retrieved from NCBI's SRA through searches during the months of November and December 2017. Samples containing metadata information indicating any type of treatment were discarded. Samples that were included had indications that they were experimental controls or did not show any metadata indicating otherwise. Expression correlation analysis of transcripts between samples was performed using the Poisson Distance and pheatmap functions of the PoiClaClu (v.1.0.2) and pheatmap (v.1.0.8) R packages. Samples that had very different behavior in relation to the majority of the same cell line or similar with different strains other than their own were discarded. That is, according to the position of the sample in the hierarchical clustering procedure and the distance. Quality control was done with FastQC (v0.11.5) and MultiQC (v1.0) with default settings. For sequence mapping, the HiSat2 (v2.0.5) aligner and the preformatted index of the reference GRCh38 release 84 of the *H. sapiens* genome from the Ensembl project was used, including dbSNP (b144) variants, splice site, and exon position information. For transcript assembly, StringTie (v1.3.1c) with strict GRCh38 annotation was used. The transcript data were tested for differential expression with the BallGown (v2.6.0) package in the R (v3.4.0) environment. A differential expression relevance cut was used for false discovery rates of less than 0.05, expression change rates greater than two, and FPKMs greater than one in at least half of the lineage samples.

### Statistical analyses

Data are presented as mean ± SD. Comparisons were performed by paired Student *t* test, one-way ANOVA with Tukey's multiple comparisons test post hoc test using GraphPad Prism 7.0 (GraphPad Software Inc., CA, USA). Significance level was *p* ≤ 0.05.

## Supplementary information

Supplemental Figure and table
