# Supplementary material for: Subverted regulation of Nox1 NADPH oxidase-dependent oxidant generation by protein disulfide isomerase A1 in colon carcinoma cells with overactivated KRas
Source: Cell Death Dis. 2019 Feb 13;10(2):143. doi: 10.1038/s41419-019-1402-y (PMC6374413; doi:10.1038/s41419-019-1402-y)
Supplement: Supplementary file 1 — Supplemental Figure and table [file 41419_2019_1402_MOESM1_ESM.docx]

**Subverted regulation of Nox1 NADPH oxidase-dependent oxidant generation by protein disulfide isomerase A1 in colon carcinoma cells with overactivated KRas**

Tiphany Coralie De Bessa ^1,2^

Alessandra Pagano ^2^

Ana Iochabel Soares Moretti ^1^

Percillia Victoria Santos de Oliveira ^1^

Samir Andrade Mendonça ^3^

Herve Kovacic ^2^*

Francisco Rafael Martins Laurindo ^1^*

**Supplemental Data:**

1. Supplementary Figure 1 to 8, with legends
2. Supplementary Table1 with legend

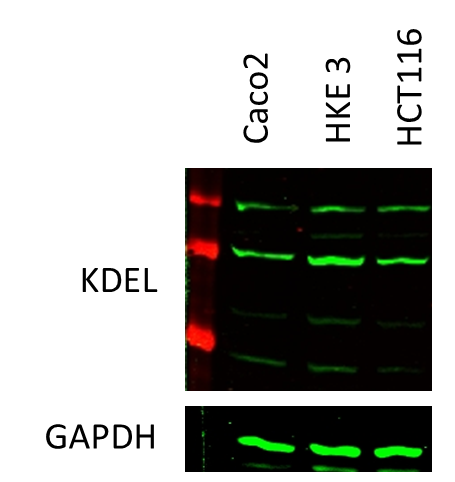


**A**

**C**

**B**

**D**


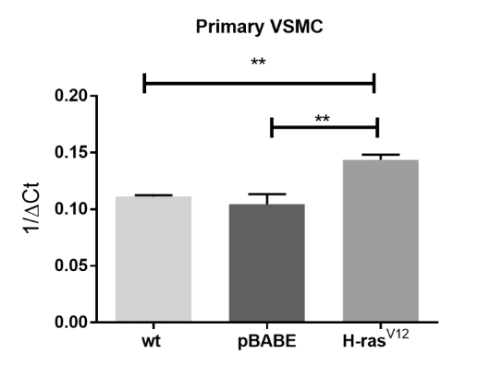


**Supplementary Figure 1:  Caracterization of colon carcinoma cells with distinct levels of KRas activation**

**(A)** KRas activity in Caco2, HKE3 and HCT116 cells. Active KRas was pulled down with GST-RBD beads from lysates of serum-starved cells treated with EGF 25ng/mL for 10min. KRas activity was quantified by KRas-GTP pull-down assay, *p<0.05, 2-way anova with Tukey's multiple comparison test (n=3). **(B)** Basal expression of KDEL-containing protein expression (ER stress marker, assayed through anti-KDEL immunoblot) in Caco2, HKE3 and HCT116 cells (n=3). **(C)** P4HB (PDIA1) gene expression :Wt: wild type VSMC; pBABE: empty vector; H-rasV12 mutant retroviral transfection in VSMC (n=3).Primers sequences are as follow for

***GAPDH****:Fw:ATGACTCTACCCACGGCAAG; Rv:CTGGAAGATGGTGATGGGTT.*

***PDIA1****:Fw:CGTGGCTACCCCACAATCA; Rv:GCTTCCCTGCCAGCTGTATATT*.

**(D)** Basal superoxide production in HKE3 and HCT116 cells, measured by HPLC analysis of DHE oxidation products (n=3).


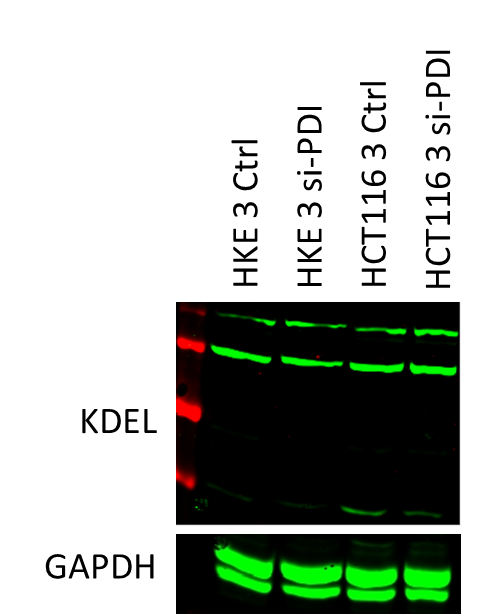


**A**

**B**

**Supplementary Figure 2:  PDIA1 silencing in Caco2, HKE3 and HCT116**

**(A)** Percentage of PDIA1 inhibition after PDIA1 silencing compared to scrambled control, assayed by immunoblot. **** *P*<0.001, Anova plus Tukey's multiple comparison test (Caco2, n=8; HKE3, n=14; HCT116, n=12). Immunoblot densities were quantified using Odyssey software. **(B)** Expression of KDEL-containing chaperones Grp78 and Grp94 in HKE3 and HCT116 72h after PDIA1 silencing (n=3).


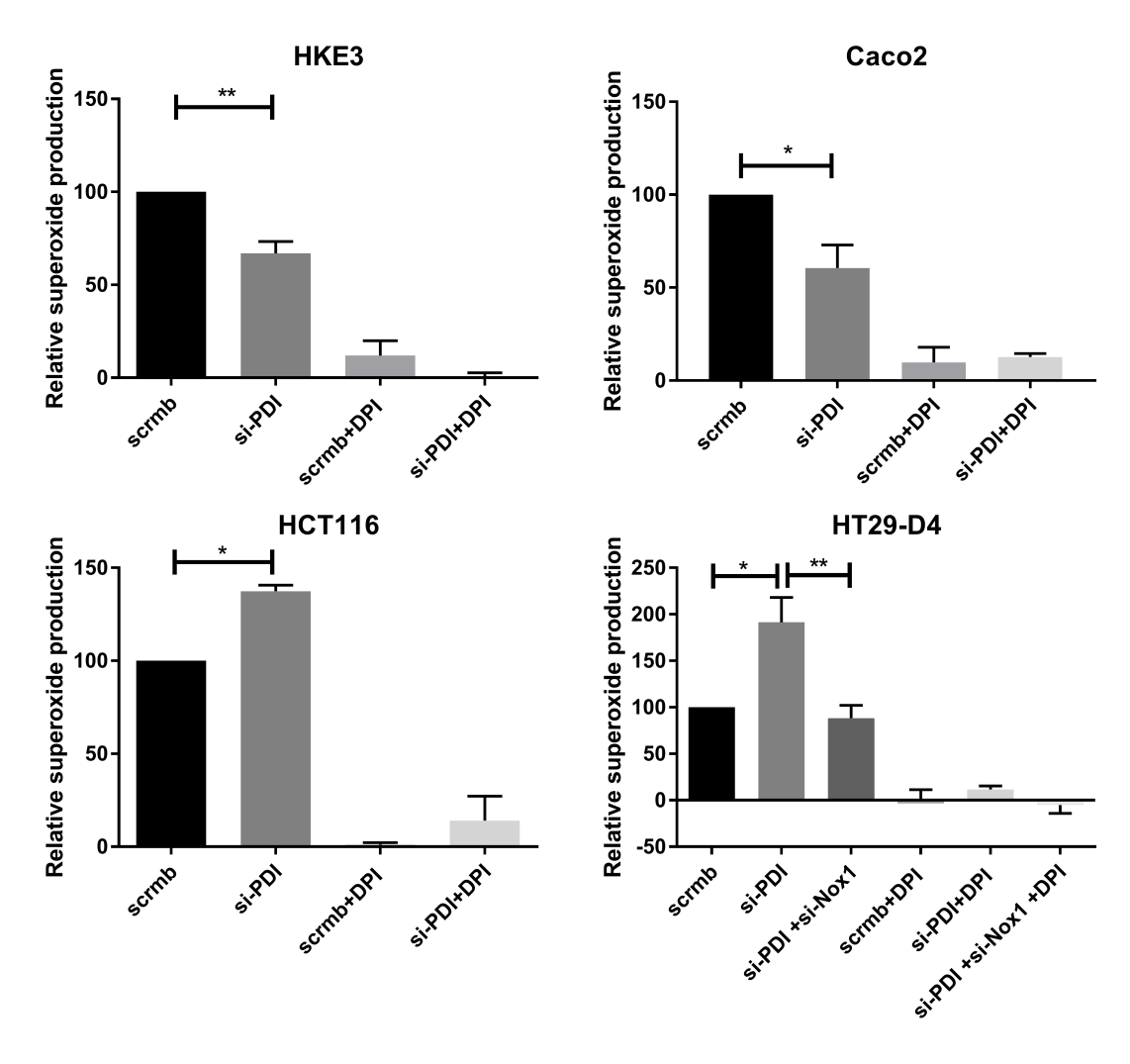


**B**

**A**

**D**

**C**

**Supplementary Figure 3 : Superoxide production in Caco2 ,HKE3, HCT116 and HT29-D4 cells After PDIA1 silencing. (A,B,C,D)** ROS (superoxide) production , measured by lucigenin oxidation assay. Scrmb : si-RNA negative control; si-PDI: si-RNA against PDIA1; scrmb+DPI: si-RNA negative control treated with 10µM of DPI (flavoprotein inhibitor); si-PDI+ DPI: si-RNA against PDIA1 treated with 10µM of DPI; si-PDI + si-Nox1: concomitant PDIA1 and Nox1 silencing; si-PDI + si-Nox1+ DPI : concomitant PDIA1 and Nox1 silencing treated with 10µM of DPI (n=3). Lucigenin oxidation assay: cells were seeded as 25 × 10^3^cells/well in 96-well plates; in the next day, cells were incubated with lucigenin and lucigenin chemiluminescence was detected by a Fluoroscan Ascent FL fluorimeter (Labsystems, France), recorded every min for 45 min.

.


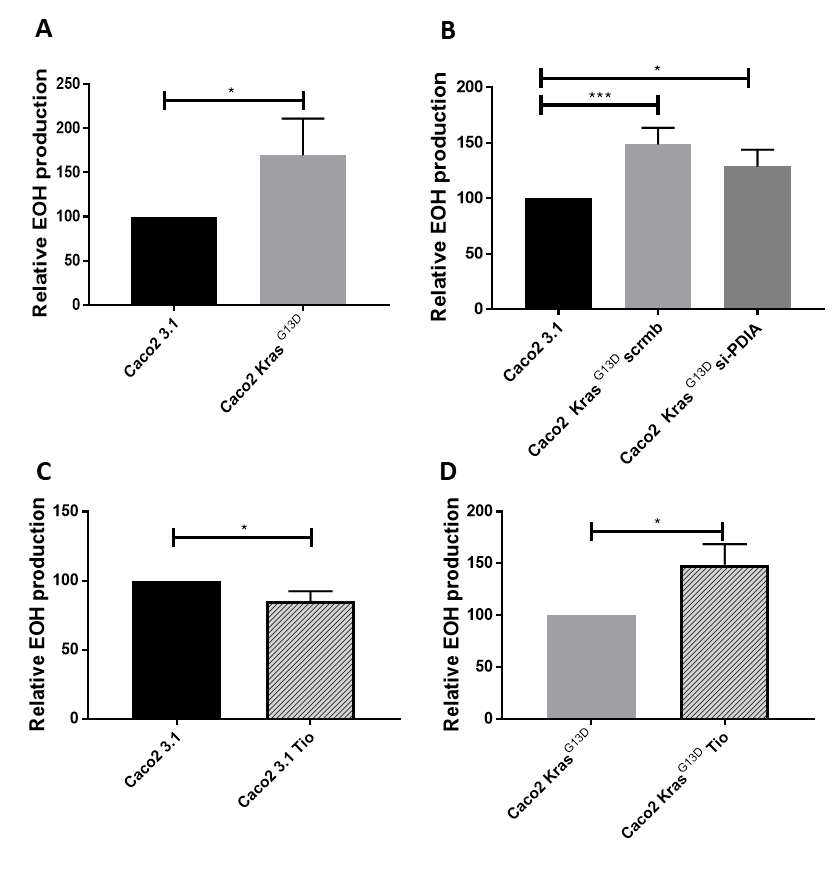


**Supplementary Figure 4: Effects of PDIA1 modulation in superoxide production in Caco2 cells after Kras^G13D^ transfection:** Superoxide was measured by HPLC analysis of DHE products. **(A)** Superoxide production in Caco2 after 72h KRas^G13D^ transfection, **Caco2 3.1**: Caco2 empty vector pCDNA3.1; **Caco2 Kras^G13D^**: Caco2 transfected with pCDNA3.1 KRas^G13D^. **p*<0.05, Student's *t* test (n=3) **(B)** Superoxide production in Caco2 after 72h of KRas^G13D^ transfection and PDIA1 silencing. **Caco2 3.1**: Caco2 empty vector pCDNA3.1; **Caco2 Kras^G13D^ scrmb**: Caco2 transfected with pCDNA3.1 KRas^G13D^and negative si-RNA control; **Caco2 Kras^G13D^ si-PDI**: Caco2 transfected with pCDNA3.1 KRas^G13D^and si-RNA against PDIA1 protein. **p*<0.05; ****P*<0.005, Anova plus Tukey's multiple comparison test (n=3). **(C-D)** Superoxide production in Caco2 48h after KRas^G13D^ transfection in the absence or presence of thiomuscimol (15µM, 15min), a thiol inhibitor acting as a non-specific but cell-permeable PDIA1 inhibitor. **Caco2 3.1**: Caco2 empty vector pCDNA3.1; **Caco2 3.1 Tio**: Caco2 empty vector pCDNA3.1 + thiomuscimol treatment; **Caco2 Kras^G13D^**: Caco2 transfected with pCDNA3.1; **Caco2 Kras^G13D^ Tio**: Caco2 transfected with pCDNA3.1 KRas^G13D^ + thiomuscimol.


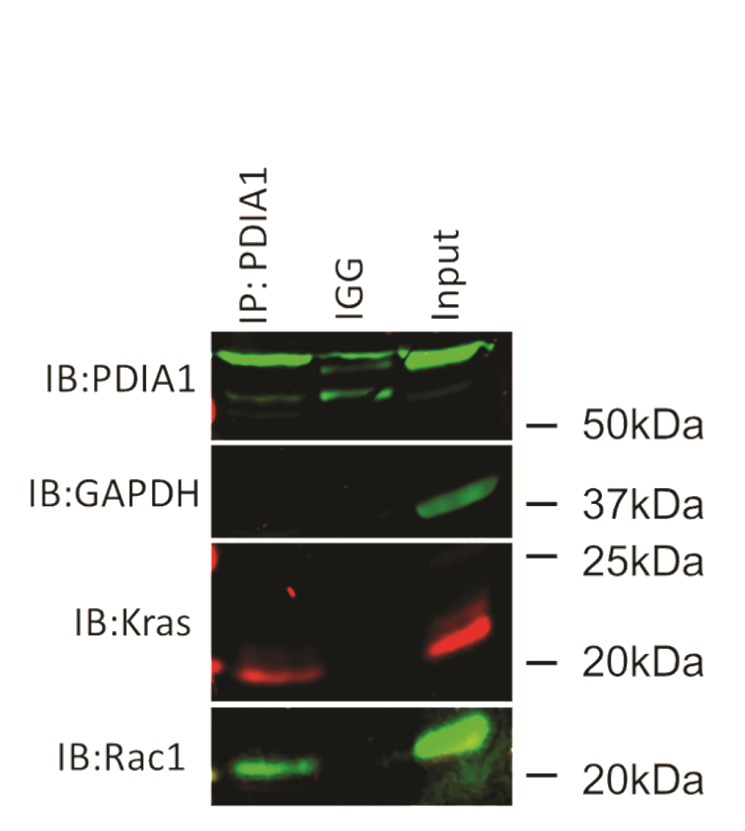


**Supplementary Figure 5: PDIA1 co-immunoprecipitation in HUVEC cells. PDIA1 co-immunoprecipitation**, IP: PDIA1 immune precipitation, IGG: Immunoglobulin control, Input: 1% of total protein lysate, IB: immunoblot against PDIA1, KRas, and Rac1 proteins. GAPDH protein expression was used as loading control, (n=1).


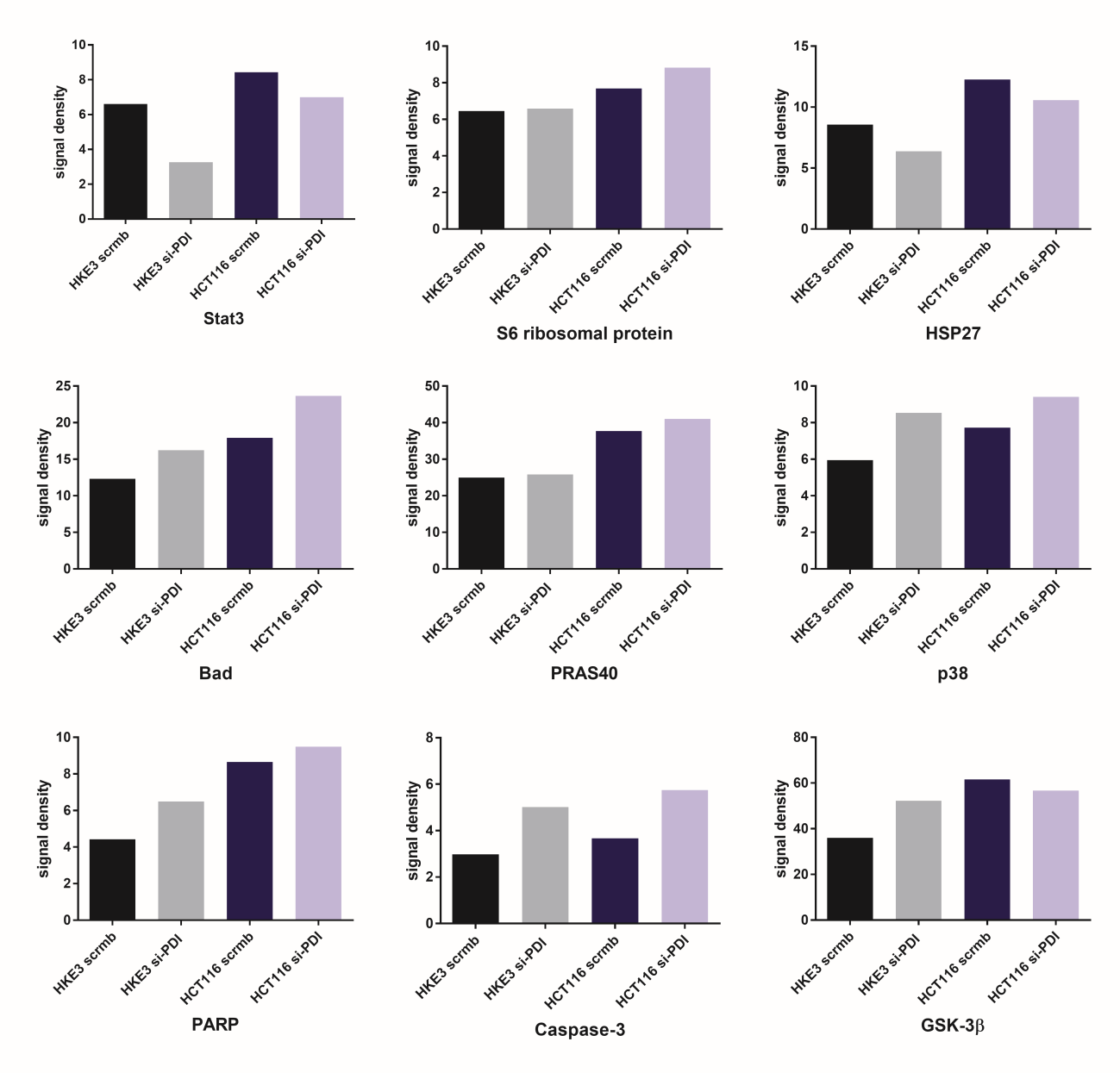


**Supplementary Figure 6: PathScan Assay screening of cell signaling targets of PDIA1 in HKE3 and HCT116 cells.**  Array’s spots were quantified and analyzed using ImageJ software. PDIA1 silencing was checked by immunoblot analysis. **Scrmb :** si-RNA negative control; **si-PDI:** si-RNA against PDIA1. **Stat3** Tyr705 Phosphorylation; **S6 Ribosomal Protein** Ser235/236 Phosphorylation; **HSP27** Ser78 Phosphorylation; **Bad** Ser112 Phosphorylation; **PRAS40** Thr246 Phosphorylation; **p38** Thr180/Tyr182 Phosphorylation; **PARP** Asp214 Cleavage; **Caspase-3** Asp175 Cleavage; **GSK-3b** Ser9 phosphorylation.


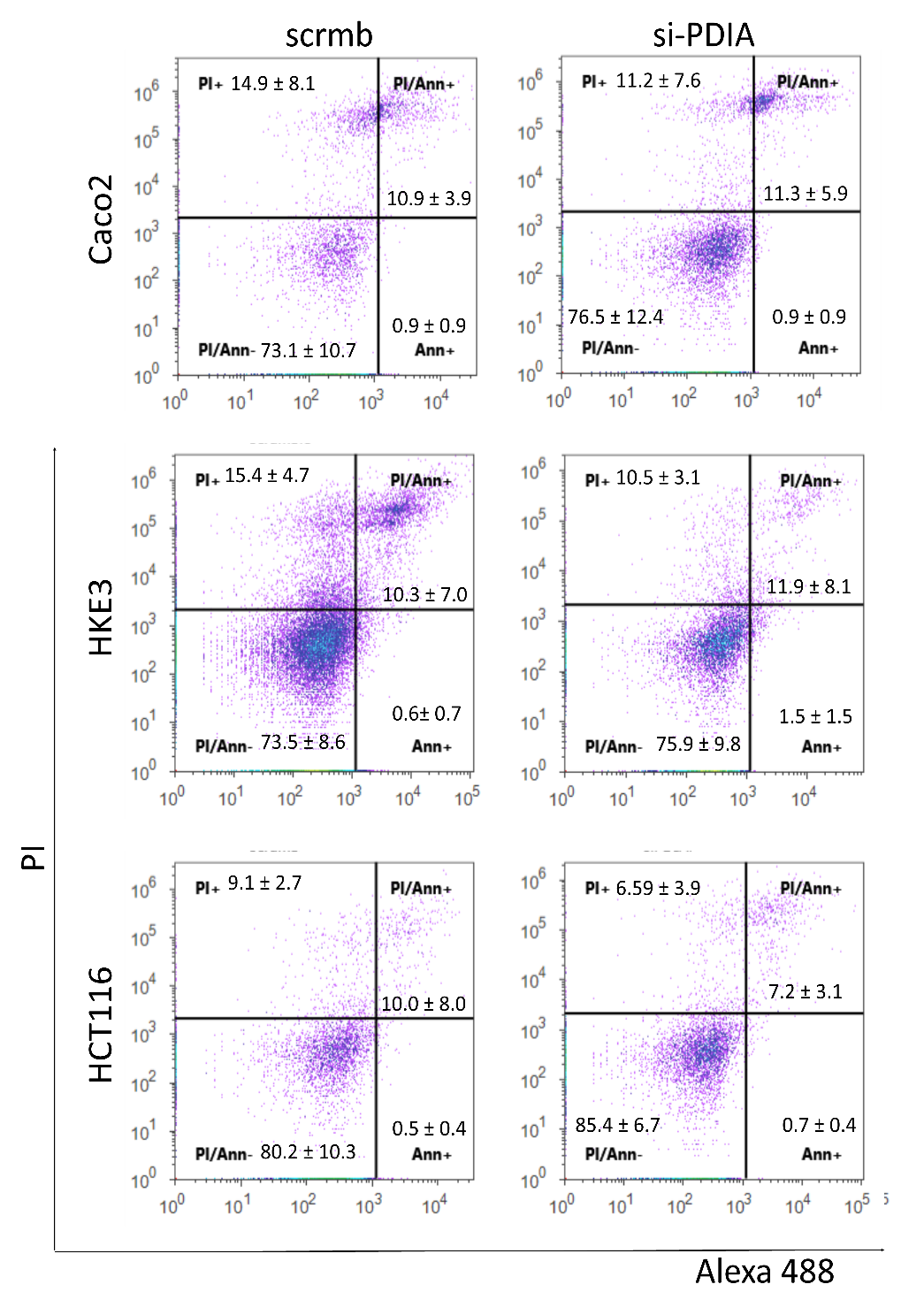

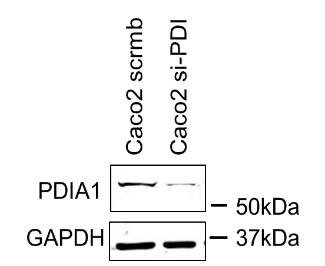

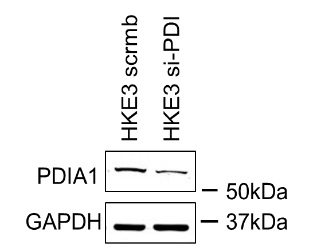

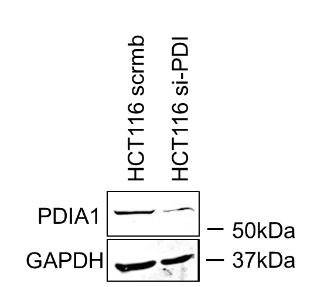


**Supplementary Figure 7: Effects of PDIA1 silencing on apoptosis:**

Representative diagram of annexin V/PI labeling by flow cytometry in Caco2, HKE3 and HCT116 cells. **Scrmb:** negative si-RNA controlsi; **PDI:** si-RNA against PDIA1 protein. Representative immunoblots of PDIA1 silencing for each cell type.


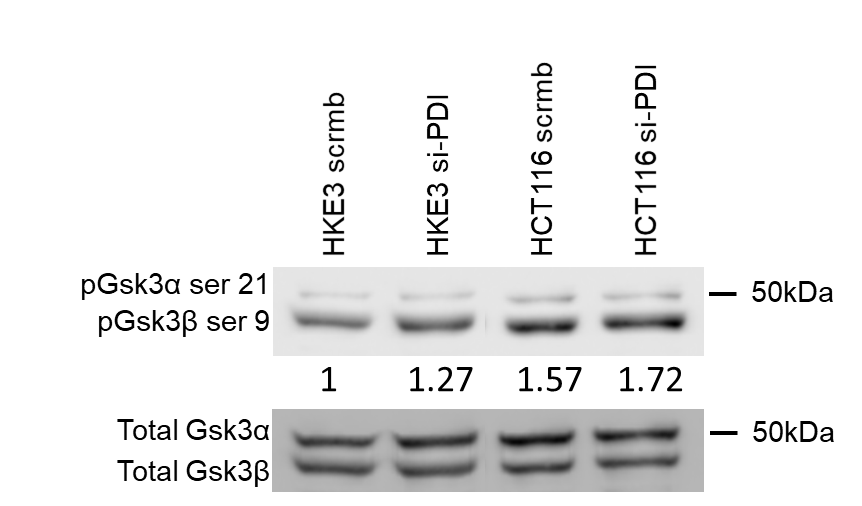


**Supplementary Figure 8: Effects of PDIA1 silencing on GSK3 inactivation in HKE3 and HCT116**. GSK3β Ser 9 phosphorylation immunoblot after 72h PDIA1 silencing in HKE3 and HCT116. Relative GSK3β Ser9 phosphorylation levels were normalized to total GSK3β expression levels. Immunoblot densities were quantified using Odyssey software.

**Supplementary Table1: Effect of PDIA1 silencing in HT29-D4 single cell migration.**

Single cell experiment, in HT29-D4 after PDIA1 silencing, HT29-D4 were plated into 24 well-plaques coated with 10mg/ml of fibronectin, in a density of 3x10^4^cells/well. Cells were maintained at 37°C in a humidified atmosphere of 5% CO2 into incubator coupled to a microscope and 2D random migration will be record for 16h. Total distance and Distance to origin are measure using image-J manual tracking. velocity as velocity= total distance/ time, and persistence as persistence= distance to origin/ total distance.

| si-PDIA1 | velocity | Total distance | Distance to origin | persistence | n=52 |
| --- | --- | --- | --- | --- | --- |
| mean | 0.33 | 199.36 | 64.65 | 0.29 |  |
| SD | 0.16 | 93.62 | 62.50 | 0.18 |  |
| SEM | 0.02 | 12.98 | 8.67 | 0.02 |  |
| si-ctrl | velocity | Total distance | Distance to origin | persistence | n=57 |
| mean | 0.33 | 182.58 | 54.72 | 0.29 |  |
| SD | 0.17 | 100.65 | 39.54 | 0.13 |  |
| SEM | 0.02 | 13.33 | 5.24 | 0.02 |  |
